# Editorial: Using Evidence Based Analytics to Create Narratives for Police Decision Making

**DOI:** 10.3389/fpsyg.2021.791605

**Published:** 2021-12-22

**Authors:** Leslie W. Kennedy, Joel M. Caplan, Simon Garnier, Kim Lersch, Fernando Miró-Llinares, Erin E. Gibbs Van Brunschot, David Lopez

**Affiliations:** ^1^School of Criminal Justice, Rutgers University, Newark, NJ, United States; ^2^Department of Biology, NJIT, Newark, NJ, United States; ^3^School of Information, University of South Florida, Tapa, FL, United States; ^4^Faculty of Social and Legal Sciences, Universidad Miquel Hernandez de Elche, Elche, Spain; ^5^Centre for Military, Security and Strategic Studies, University of Calgary, Calgary, AB, Canada; ^6^Rutgers Law School, Rutgers, The State University of New Jersey, Newark, NJ, United States

**Keywords:** police decision making, narratives, evidence based, hot spots, analytics

Pressure for reform of modern policing has come from the combined demands of greater accountability, transparency and citizen demand for more culturally sensitive responses. The community policing strategies that have emerged from these reforms have led to an increased interest in the ways that police manage decision making when applying principles of crime control and order maintenance. In particular, it has been observed that decision making is directly influenced by greater access to data and more sophisticated analysis tools. As a consequence, it is possible to monitor all aspects of police activity including interactions with the public. This has led to increased pressure on agencies to develop protocols that mandate awareness of procedural justice into police training. Also, it has led to outreach to community members to engage them in planning strategies for resource allocation and crime control. In developing this new paradigm of community involvement, law enforcement has come to rely on crime analysts and criminologists in the collaborative decision making process.

This introduction of data analytics into the mix has created new challenges in the operational world of policing. In particular, the outcomes of data analysis can be highly abstract and go counter to the intuition or experiences of police officers. This can leave analysts struggling to find common ground between the evidence that is presented through their analysis and the beliefs of members. To overcome this tension, we need to increase our understanding of the bases on which police justify their best practices and their impacts in prevention and control. Broadening beyond individual intuition and increasing our understanding of what effective practices can lead to better outcomes is an imperative that is a new challenge for police researchers and analysts.

The papers included here aim to explore the varying components of crime analysis and forecasting that influence police decision making, as discussed above. They emphasize how evidence-based approaches that combine individual, spatial, and temporal data can improve their daily decision making processes. In the papers included here, the authors have approached different aspects of this inquiry, providing important guidance of how we might explore these questions in future work.

Berryessa and Caplan look at how different types of data-derived information about crime and safety risks, such as maps, graphs or tables, uniquely influence decision-making and actions by police. Results showed that risk information is read by people to create their own dichotomous distinctions of safety or danger that inform their actions, even when multiple categories of variation from low-to-high risk existed. This study suggests that maps, graphs or tables communicating low-crime areas, or “cold spots,” could be more intuitively meaningful for police and other public safety stakeholders.

The paper by Shortland et al. delves into the aspects of police decision making that reflects the experience of officers involved in these processes. It suggests that there are major differences in the ways in which personal background and experience influence how officers make decisions that guide their actions. Walczak explores how neural networks form the decisions of officers working in evidence based approaches. Gerell et al. round out the collection with a real world example of how the Swedish police operated a helicopter surveillance as a substitute to car patrols. These papers offer different perspectives that demonstrate the versatility of evidence based approaches and the ways that data can inform narratives among agents of social control.

Future work should explore these themes in more detail in establishing how we can improve on group practice and make police decision making more deliberative and concise. The importance of this topic has been demonstrated in the recent growth of professional cross disciplinary organizations that have promoted the investigation of evidence based approaches. Work on improving responses to mental health crises, for example, has promoted the idea that a better understanding of the underlying causes of these incidents would assist in redefining decisions that are made by agencies on the ground responding to them. The efforts that have been in place in public health, psychology and medicine have slowly been replicated in criminology and criminal justice. We hope that this collection will contribute to the ways in which these academics and practitioners attracted to these groups develop their inquiries and form future research agendas around this topic.

## Author Contributions

All co-authors recognize LK leadership and substantial and direct contribution to this publication. All authors intellectually contributed to this work and approved it for publication.

## Conflict of Interest

The authors declare that the research was conducted in the absence of any commercial or financial relationships that could be construed as a potential conflict of interest.

## Publisher's Note

All claims expressed in this article are solely those of the authors and do not necessarily represent those of their affiliated organizations, or those of the publisher, the editors and the reviewers. Any product that may be evaluated in this article, or claim that may be made by its manufacturer, is not guaranteed or endorsed by the publisher.

